# The Dual RAF/MEK Inhibitor CH5126766/RO5126766 May Be a Potential Therapy for RAS-Mutated Tumor Cells

**DOI:** 10.1371/journal.pone.0113217

**Published:** 2014-11-25

**Authors:** Makoto Wada, Mano Horinaka, Toshikazu Yamazaki, Norito Katoh, Toshiyuki Sakai

**Affiliations:** 1 Department of Molecular-Targeting Cancer Prevention, Graduate School of Medical Science, Kyoto Prefectural University of Medicine, Kyoto, Japan; 2 Department of Dermatology, Graduate School of Medical Science, Kyoto Prefectural University of Medicine, Kyoto, Japan; 3 Kamakura Research Laboratory, Research Division, Chugai Pharmaceutical Co., Kamakura, Japan; University of Queensland Diamantina Institute, Australia

## Abstract

Although melanoma is the most aggressive skin cancer, recent advances in BRAF and/or MEK inhibitors against BRAF-mutated melanoma have improved survival rates. Despite these advances, a treatment strategy targeting NRAS-mutated melanoma has not yet been elucidated. We discovered CH5126766/RO5126766 as a potent and selective dual RAF/MEK inhibitor currently under early clinical trials. We examined the activity of CH5126766/RO5126766 in a panel of malignant tumor cell lines including melanoma with a BRAF or NRAS mutation. Eight cell lines including melanoma were assessed for their sensitivity to the BRAF, MEK, or RAF/MEK inhibitor using *in vitro* growth assays. CH5126766/RO5126766 induced G1 cell cycle arrest in two melanoma cell lines with the BRAF V600E or NRAS mutation. In these cells, the G1 cell cycle arrest was accompanied by up-regulation of the cyclin-dependent kinase inhibitor p27 and down-regulation of cyclinD1. CH5126766/RO5126766 was more effective at reducing colony formation than a MEK inhibitor in NRAS- or KRAS-mutated cells. In the RAS-mutated cells, CH5126766/RO5126766 suppressed the MEK reactivation caused by a MEK inhibitor. In addition, CH5126766/RO5126766 suppressed the tumor growth in SK-MEL-2 xenograft model. The present study indicates that CH5126766/RO5126766 is an attractive RAF/MEK inhibitor in RAS-mutated malignant tumor cells including melanoma.

## Introduction

The prognosis of patients with metastatic melanoma is poor with a 5-year survival rate of less than 5%, which reflects the failure of chemotherapy and immunotherapy regimens [1]. Although dacarbazine has been the standard first-line chemotherapy for decades, new therapies such as molecular-targeting agents against BRAF-mutated melanoma or the anti-CTLA4 monoclonal antibody ipilimumab have improved the survival rate [2]–[7]. In the majority of human melanomas, the mitogen-activated protein kinase (MAPK) pathway is constitutively activated by oncogenic mutations in NRAS or BRAF [8]–[10]. BRAF inhibitors such as vemurafenib (PLX4032, RG7204) and dabrafenib (GSK2118436) cause marked responses in patients with BRAF-mutant melanoma, but not in patients with BRAF wild-type melanoma [4], [5]. The novel MEK inihibitor trametinib, which we discovered by screening to detect p15-inducing agents [11], improved progression-free and overall survival in patients with BRAF V600E-mutated metastatic melanoma [6], and was approved by FDA in 2013. In addition, the combination of the BRAF inhibitor dabrafenib and the MEK inhibitor trametinib improved progression-free survival in patients with BRAF V600E-mutated metastatic melanoma [7]. However, BRAF inhibitors also paradoxically activated the MEK/ERK pathway in cells expressing oncogenic RAS [12]–[15]. In phase I study of salirasib, a RAS inhibitor, in patients with solid tumor, 7 of 24 patients had stable disease for 4 months or longer (range 4–13 months) [16]. However, in phase II trial of salirasib in patients with lung adenocarcinoma with KRAS mutations, 7 of 23 patients had stable disease for 10 weeks, but no radiographic partial response was observed [17]. On the other hand, similar to the discovery of trametinib, we found the novel RAF/MEK inhibitor CH5126766/RO5126766 by screening to detect p27-inducing agents [18]. CH5126766 has the unique property of inhibiting RAF kinase. RAF tightly binds to MEK, and CH5126766 then binds to MEK, such that RAF cannot be phosphorylated and released [18], [19].

Here we show that the novel RAF/MEK inhibitor CH5126766 suppresses the cell growth of RAS-mutated cells as well as BRAF-mutated cells, which raises the possibility that CH5126766 is promising for the therapy against RAS-mutated malignant tumors.

## Materials and Methods

### Cell culture

SK-MEL-28, SK-MEL-2, A549, HCT15, HCT116, SW480, and PC3 cells were obtained from the American Type Culture Collection. MIAPaCa-2 cells were obtained from Health Science Research Resources Bank. All cell lines were expanded and placed in stock within a month of receipt. SK-MEL-28, SK-MEL-2, MIAPaCa-2, and A549 cells were maintained in DMEM. SW480, HCT15, HCT116, and PC3 cells were maintained in RPMI 1640. Culture media were supplemented with 10% fetal bovine serum, L-glutamine (2 mM for RPMI 1640 and 4 mM for DMEM), 50 U/mL penicillin, and 100 µg/mL streptomycin. Cell cultures were incubated at 37°C in a humidified atmosphere of 5% CO_2_.

### Reagents

CH5126766, PD0325901, and PLX4720 were provided by Chugai Pharmaceutical Co., Ltd. All compounds were dissolved in dimethyl sulfoxide (DMSO) as stock and stored at −80°C.

### Cell viability assay

The number of viable cells was determined using the Cell Counting Kit-8 assay according to the manufacturer's instructions (Dojindo). After the incubation of cells for 72 h with the indicated concentrations of various agents, kit reagent WST-8 was added to the medium and incubated for a further 4 h. The absorbance of samples (450 nm) was determined using a scanning multiwell spectrophotometer that serves as an ELISA reader. Cell numbers and viability were also measured using the ViaCount Assay according to the manufacturer's instructions (Guava Technologies).

### Cell cycle and apoptosis analyses

Cells were incubated with or without agents as indicated, and harvested. They were then trypsinized, and stained with 100 µg/mL of propidium iodide. Flow cytometry was carried out with a FACScalibur (Becton-Dickinson). DNA fragmentation was quantified on the basis of the percentage of hypodiploid DNA (sub-G1). Data were analysed with CellQuest software (Becton Dickinson) and Modfit software (Verity Software House).

### Western blot analysis

Cells were incubated with or without agents as indicated, and harvested. The cells were then re-suspended in lysis buffer (50 mM Tris-HCl, 1% SDS, 2 µg/mL leupeptin, 2 µg/mL aprotinin, 0.5 mM phenylmethylsulfonyl fluoride, and 0.1% 2-mercaptoethanol). The lysate was sonicated and centrifuged at 15,000 g for 20 min at 4°C, and the supernatant was collected. Equal amounts of lysate were analysed by SDS-PAGE and transferred to PVDF membranes (Millipore). Primary antibodies were obtained for the following proteins: MEK1/2 (#9122), phospho-MEK1/2 Ser217/221 (#9121), p44/42 MAPK (ERK1/2) (#9102), phospho-p44/42 MAPK (ERK1/2) Thr202/Tyr204 (#9101), phospho-RB Ser780 (#9307), phospho-RB Ser807/811 (#9308) (Cell Signaling Technology, Inc.), p27 (sc-528), cyclinE (sc-247), Raf-1 (CRAF) (sc-7267), Raf-B (BRAF) (sc-55522) (Santa Cruz Biotechnology, Inc.), β-actin (Sigma), RB (BD Bioscience), and cyclinD1 (K0062-3) (Medical and Biologic Laboratories). The blots were blocked in blocking buffer (5% skim milk/TBST) for 1 h at room temperature, and incubated with the appropriate primary antibody in blocking buffer for 1 h at room temperature. The blots were then washed and incubated with the appropriate horseradish peroxidase (HRP)-conjugated secondary antibody for 1 h, and signals were detected with the Immobilon Western Chemiluminescent HRP Substrate (Millipore).

### Colony formation assay

Cells were seeded at a density of 100 cells in each well of the 6-well plates. The cells were treated with each agent for 3 days. After 3 days, agent-treated cells were then washed twice in PBS followed by the addition of fresh media. After further incubation for 10-14 days, cells were fixed with 10% formalin, and stained with 0.1% crystal violet in 20% ethanol.

### Small interfering RNA transfection

Small interfering RNAs (siRNA) were obtained from Invitrogen for BRAF (5′-AAUAGGGCCUCUAUAUGUUCCUGUG-3′), CRAF (5′-CAGCUGCAUCUCUCCUACAAUAGUU-3′), and Stealth^TM^ RNAi Negative Control Duplexes. Cells were transfected with 10 nM of each siRNA using the Invitrogen RNAi MAX reagent. Twenty-four h after the transfection, cells were incubated with CH5126766, PD0325901, or 0.1% DMSO for the indicated time, and harvested for immunoblotting.

### Mouse xenograft models

Animal xenograft studies were approved by the Chugai Institutional Animal Care and Use Committee. Female BALB-nu/nu mice (CAnN.Cg-Foxn1nu/CrlCrlj nu/nu) were obtained from Charles River Laboratories Japan and maintained under pathogen-free conditions. These mice were given access to standard mouse chow and water ad libitum. A total of 1×10^7^ SK-MEL-2 cells were injected subcutaneously into the right inguinal area of 7–9-week-old mice. When the tumor volume reached 200 mm^3^ (day 0), the mice were randomized to each group (n = 5) and vehicle or CH5126766 was orally administered once a day. Statistical analysis was conducted by using Dunnett test. The criterion for statistical significance was P<0.05.

### Statistical analysis

Data were expressed as the mean ± SD of three determinations. Significance was assessed by a Student's *t*-test *in vitro* analysis, or by a Dunnett test *in vivo* analysis. A value of *P*<0.05 was considered to be significantly different from DMSO-treated controls.

## Results

### *In vitro* activity of MEK and RAF inhibitors

To investigate the antiproliferative effects of MEK and RAF inhibitors, we used the dual RAF/MEK inhibitor CH5126766, MEK inhibitor PD0325901, and RAF inhibitor PLX4720. We employed eight cancer cell lines including two melanoma with various mutations such as RAS, RAF, PIK3CA, and PTEN (Table 1). For each cell line, the IC_50_ was determined by a WST-8 assay under a 72 h continuous exposure to CH5126766, PD0325901, or PLX4720. A cell line containing the BRAF V600E mutation was sensitive to CH5126766, PD0325901, or PLX4720, whereas cells containing the NRAS or KRAS mutation with wild-type BRAF were variably sensitive to CH5126766 or PD0325901, and insensitive to PLX4720. PC3 cells with PTEN mutation were resistant to CH5126766, PD0325901, and PLX4720.

**Table 1:** Effects of RAF, MEK, and RAF/MEK inhibitors on cancer cell lines with or without RAS, BRAF, PIK3CA, or PTEN mutations.

Cell line	Tissue	RAS	RAF	PIK3CA/PTEN	CH	PD	PLX
SK-MEL-28	melanoma	WT	BRAF(V600E)	WT	65	4.3	345
SK-MEL-2	melanoma	NRAS(Q61R)	WT	WT	28	4.5	>1000
MIAPaCa-2	pancreas	KRAS(G12C)	WT	WT	40	1.5	>1000
SW480	colon	KRAS(G12V)	WT	WT	46	25	>1000
A549	lung	KRAS(G12S)	WT	WT	>1000	>1000	>1000
HCT15	colon	KRAS(G13D)	WT	PIK3CA(E545K)	>1000	>1000	>1000
HCT116	colon	KRAS(G13D)	WT	PIK3CA(H1047R)	277	47	>1000
PC3	prostate	WT	WT	PTEN(del)	>1000	>1000	>1000

CH; CH5126766, PD; PD0325901, PLX; PLX4720 IC_50_ (nM).

**Figure 1:**
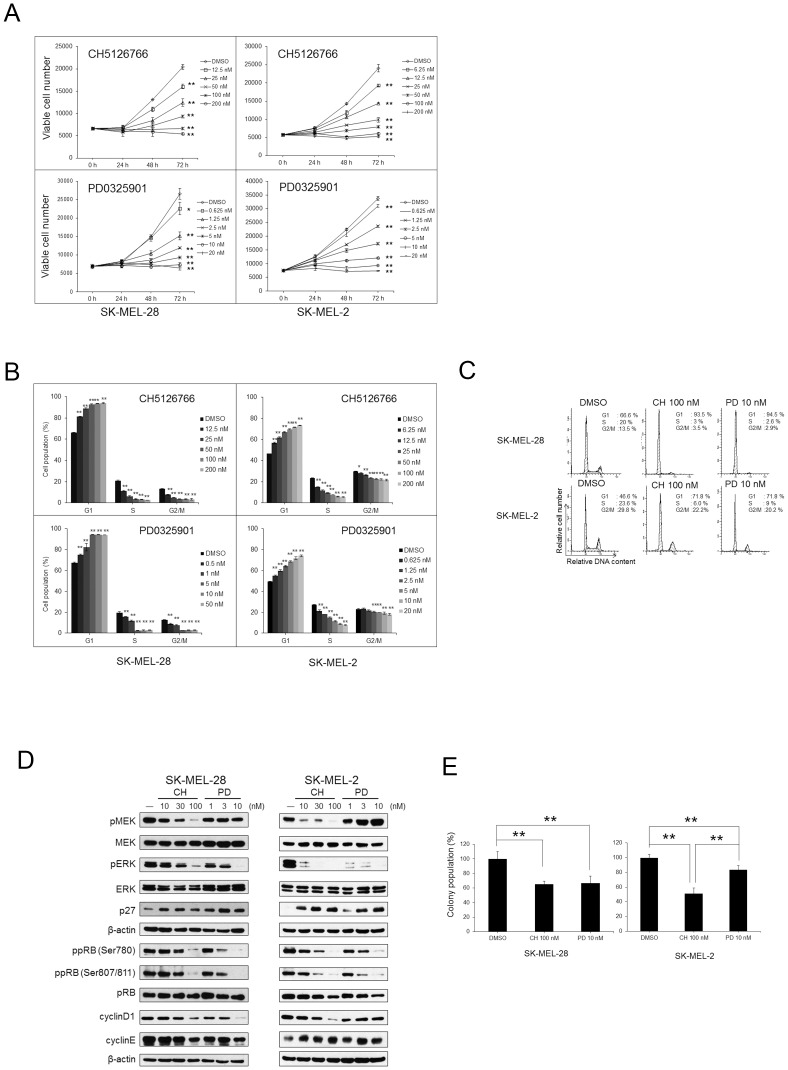
Effects of the RAF/MEK inhibitor CH5126766 and the MEK inhibitor PD0325901 on melanoma cell lines. A, SK-MEL-28 and SK-MEL-2 cells were treated with CH5126766 or PD0325901 at the various concentrations, and cell numbers were determined using a Guava EasyCyte plus flow cytometer. Data represent the means of triplicate with SD indicated. Significance was assessed by a Student's *t* test. *, P<0.05; **, P<0.01 significantly different from DMSO-treated controls. B, SK-MEL-28 and SK-MEL-2 cells were treated with the indicated concentrations of CH5126766 or PD0325901 for 24 h. The percentage of cells in each phase of the cell cycle was determined by flow cytometry. Data represent means of triplicate with SD indicated. Statistical significance was assessed by a Student's *t* test. *, P<0.05; **, P<0.01 compared with DMSO-treated controls. C, Representative histogram patterns from SK-MEL-28 and SK-MEL-2 cells. D, SK-MEL-28 and SK-MEL-2 cells were treated with the indicated concentrations of CH5126766 or PD0325901 for 24 h, and cell extracts were analysed by immunoblotting. E, Clonogenic suppression by CH5126766 and PD0325901 in melanoma cells. SK-MEL-28 and SK-MEL-2 cells were treated for 72 h with 10 nM of PD0325901 or 100 nM of CH5126766. After 14 days, colony numbers were counted. Data represent the means of triplicate with SD indicated. Significance was assessed by a Student's *t* test. **, P<0.01. CH; CH5126766, PD; PD0325901

To examine the antiproliferative effects of CH5126766 or PD0325901, we used two melanoma cell lines with mutations resulting in ERK activation, SK-MEL-28 (BRAF V600E), and SK-MEL-2 (NRAS Q61R). As shown in Figure 1A, both inhibitors inhibited the growth of two cell lines in dose- and time-dependent manners. We next investigated the effects of both inhibitors on cell cycle progression and apoptosis in two cell lines by flow cytometry analysis. As shown in Figure 1B and 1C, CH5126766 or PD0325901 induced G1 cell cycle arrest in two cell lines. Both inhibitors were unable to induce apoptosis in either cell line at 24 h after the treatment (Figure S1). In addition, CH5126766 could not sufficiently induce apoptosis even at 72 or 96 h after the treatment (data not shown). We then investigated the mechanism of G1 cell cycle arrest by Western blotting. As shown in Figure 1D, we found that the phosphorylation of MEK was inhibited by CH5126766 at a concentration of 10 nM or more in both cell lines due to its RAF-inhibitory activity. In contrast, phospho-MEK levels were significantly increased in SK-MEL-2 cells treated with PD0325901. The accumulation of phospho-MEK after the treatment with PD0325901 may be due to the abrogation of negative feedback between ERK and upstream targets in NRAS-mutated cells [20]. CH5126766 and PD0325901 effectively reduced phospho-ERK and increased the expression of p27 in both cell lines. In contrast, CH5126766 more effectively decreased cyclinD1 expression than PD0325901 in SK-MEL-2 cells, whereas CH5126766 and PD0325901 similarly inhibited cyclinD1 expression in SK-MEL-28 cells (Fig. 1D). Colony formation assays were conducted to evaluate prolonged inhibitory effects on cell growth using 100 nM CH5126766 or 10 nM PD0325901, which have shown the same cytostatic inhibition in Figure 1A. Figure 1E shows that both inhibitors exhibited the same degree of inhibition in colony population in SK-MEL-28 cells with the BRAF mutation. However, the RAF/MEK inhibitor CH5126766 was more effective at reducing colony formation than the MEK inhibitor PD0325901 in SK-MEL-2 cells with the NRAS mutation.

### The RAF inhibitor negated MEK inhibition by PD0325901 in the RAS-mutated cell line

**Figure 2:**
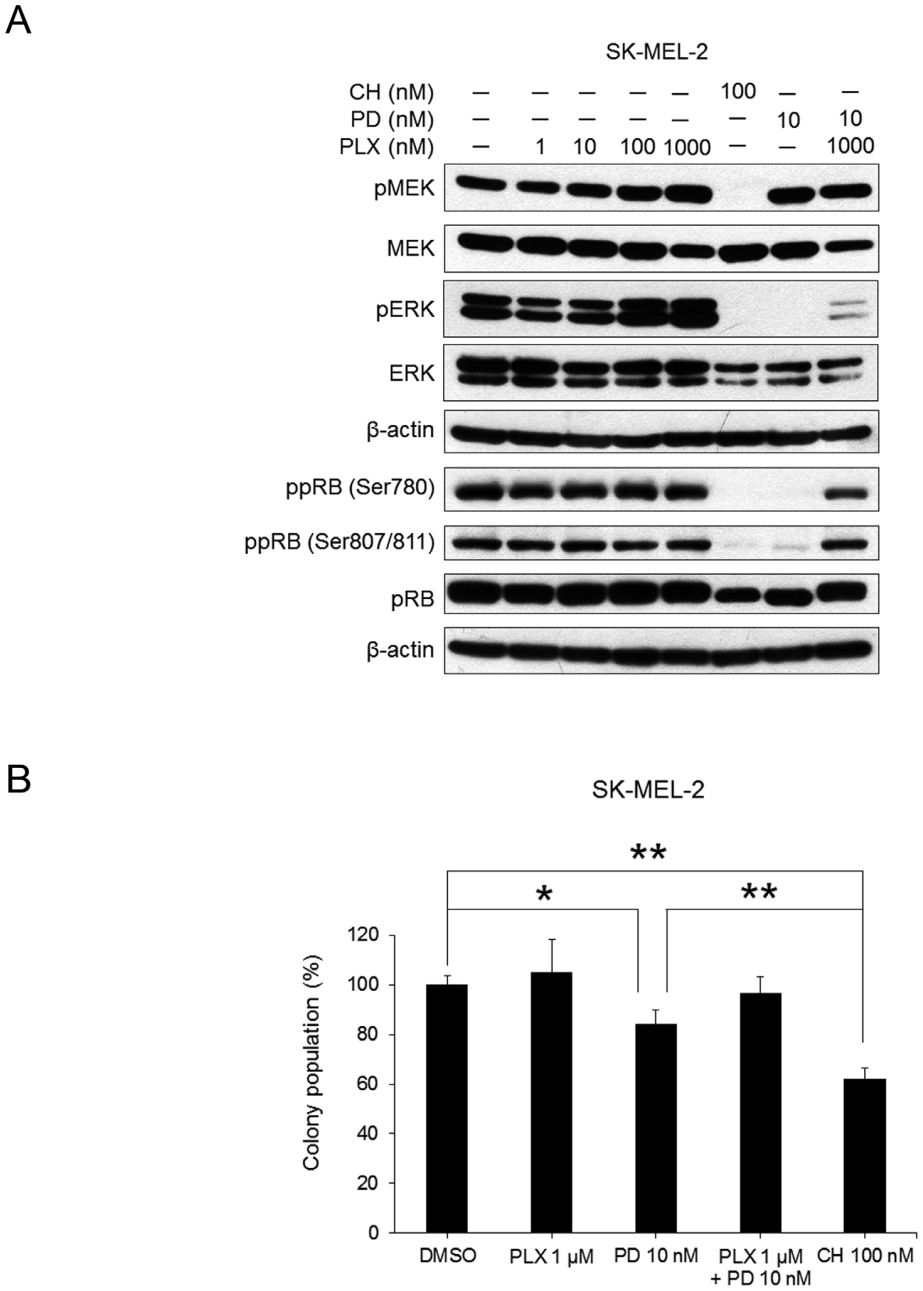
The RAF inhibitor PLX4720 negated MEK inhibition by PD0325901 in a NRAS-mutated cell line. A, SK-MEL-2 cells were treated with PLX4720 in the presence or absence of CH5126766 or PD0325901 for 24 h. Cell extracts were analysed by immunoblotting for the indicated proteins. B, Clonogenic suppression by PD0325901 and/or PLX4720 in NRAS-mutated SK-MEL-2 cells. SK-MEL-2 cells were treated for 72 h with 100 nM of CH5126766, or 10 nM of PD0325901 in the presence or absence of 1 µM of PLX4720. After 14 days, colony numbers were counted. Data represent the means of triplicate with SD indicated. Significance was assessed by a Student's *t* test. *, P<0.05; **, P<0.01. CH; CH5126766, PD; PD0325901, PLX; PLX4720

To investigate whether the RAF inhibitor could suppress the accumulation of phospho-MEK after treatment with the MEK inhibitor, NRAS-mutated SK-MEL-2 cells were simultaneously treated with the RAF inhibitor PLX4720 and the MEK inhibitor PD0325901. The treatment with PLX4720 in SK-MEL-2 cells resulted in an increase in the phosphorylation of MEK and ERK. PLX4720 abrogated the suppression of phosphorylated ERK and RB by PD0325901 in SK-MEL-2 cells (Fig. 2A). We then evaluated the effect of PLX4720 and/or PD0325901 on cell growth of SK-MEL-2 cells by colony formation assay. As shown in Figure 2B, the combined treatment of PLX4720 and PD0325901 did not significantly reduce the colony formation, whereas PD0325901 only significantly inhibited the colony formation of SK-MEL-2 cells. PLX4720 reduced the effect of PD0325901 in NRAS-mutated melanoma cells (Fig. 2A), whereas the RAF/MEK inhibitor CH5126766 markedly inhibited the colony formation with inhibition of the RAF-MEK pathway even in NRAS-mutated melanoma cells (Fig. 1E, Fig. 2A).

### Effects of MEK or RAF/MEK inhibitor in KRAS-mutated cell lines

**Figure 3:**
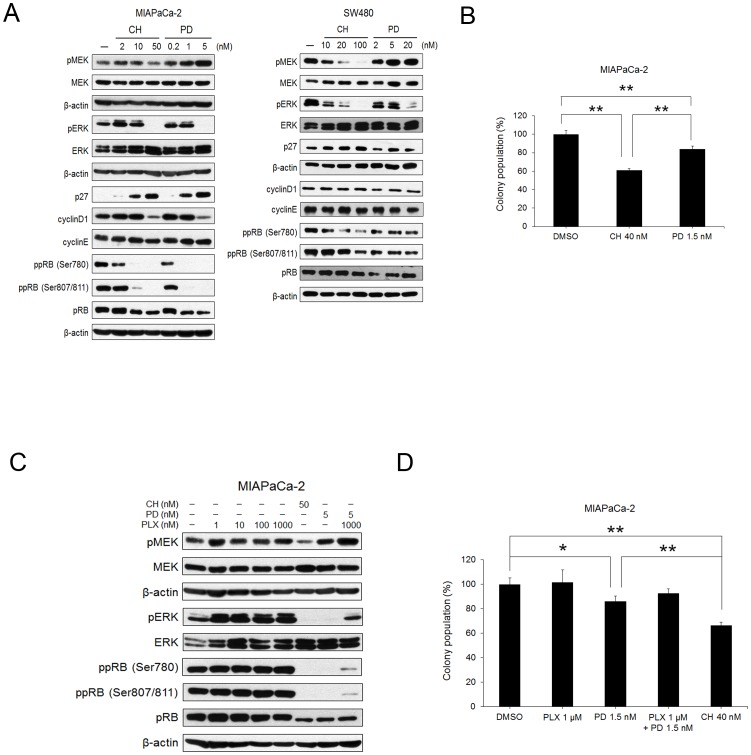
Effects of the RAF/MEK inhibitor CH5126766 and the MEK inhibitor PD0325901 on KRAS-mutated cell lines. A, MIAPaCa-2 and SW480 cells were treated with CH5126766 or PD0325901 at the indicated concentrations for 24 h, and cell extracts were analysed by immunoblotting for the indicated proteins. B, Clonogenic suppression by CH5126766 or PD0325901 in MIAPaCa-2 cells. MIAPaCa-2 cells were treated for 72 h with 40 nM of CH5126766 or 1.5 nM of PD0325901. After 14 days, colony numbers were counted. Data represent the means of triplicate with SD indicated. Significance was assessed by a Student's *t* test. **, P<0.01. C, MIAPaCa-2 cells were treated with CH5126766 or PD0325901 in the presence or absence of PLX4720 at the indicated concentrations for 24 h, and cell extracts were analysed by immunoblotting for the indicated proteins. D, Clonogenic suppression by PD0325901 and/or PLX4720 in MIAPaCa-2 cells. MIAPaCa-2 cells were treated for 72 h with 40 nM of CH5126766, or 1.5 nM of PD0325901 in the presence or absence of 1 µM of PLX4720. After 14 days, colony numbers were counted. Data represent the means of triplicate with SD indicated. Significance was assessed by a Student's *t* test. *, P<0.05; **, P<0.01. CH; CH5126766, PD; PD0325901, PLX; PLX4720

Moreover, we examined two additional KRAS-mutated cell lines, the pancreatic cancer cell line MIAPaCa-2 and colorectal cancer cell line SW480. The phosphorylation of MEK was inhibited by CH5126766, and increased by PD0325901 in both cell lines. Both inhibitors decreased phospho-RB in both cell lines, but decreased cyclinD1 and increased p27 in MIAPaCa-2, but not in SW480 cells (Fig. 3A). By a colony formation assay using 40 nM of CH5126766 or 1.5 nM of PD0325901, which were the same IC50 concentrations as the WST-8 assay (Table 1), CH5126766 more efficiently inhibited colony formation than PD0325901 (Fig. 3B). Colonies were not able to be formed on the plate dish in SW480 cells. PLX4720 abrogated the inhibition of phospho-ERK by PD0325901 in MIAPaCa-2 cells, similar to SK-MEL-2 cells (Fig. 3C). Consistently, the combined treatment of PLX4720 and PD0325901 did not significantly reduce the colony formation, whereas PD0325901 only significantly inhibited the colony formation of MIAPaCa-2 cells (Fig. 3D).

### RAS required CRAF to activate the MEK/ERK pathway

**Figure 4:**
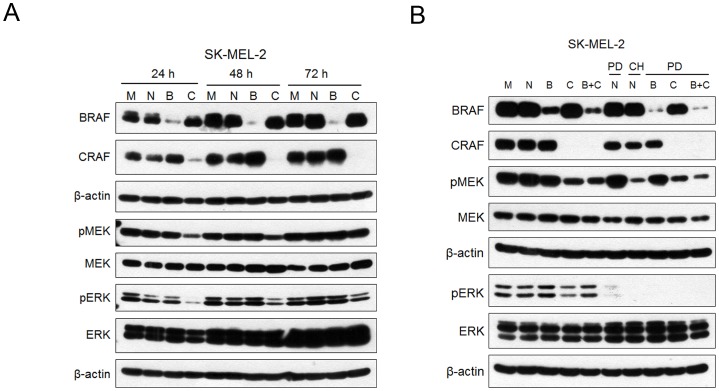
Oncogenic NRAS required CRAF to activate the MEK/ERK pathway. A, SK-MEL-2 cells were transfected with siRNAs for the negative control (N), BRAF (B), or CRAF (C), and without siRNAs for mock (M). After the indicated times, cell extracts were analysed by immunoblotting for the indicated proteins. B, SK-MEL-2 cells were transfected with siRNA for the negative control (N), BRAF (B), and CRAF (C) , and without siRNAs for mock (M). After 24 h, 100 nM of CH5126766 or 10 nM of PD0325901 were added to the cells. The cells were harvested for immunoblot analysis after 24 h. CH; CH5126766, PD; PD0325901

**Figure 5:**
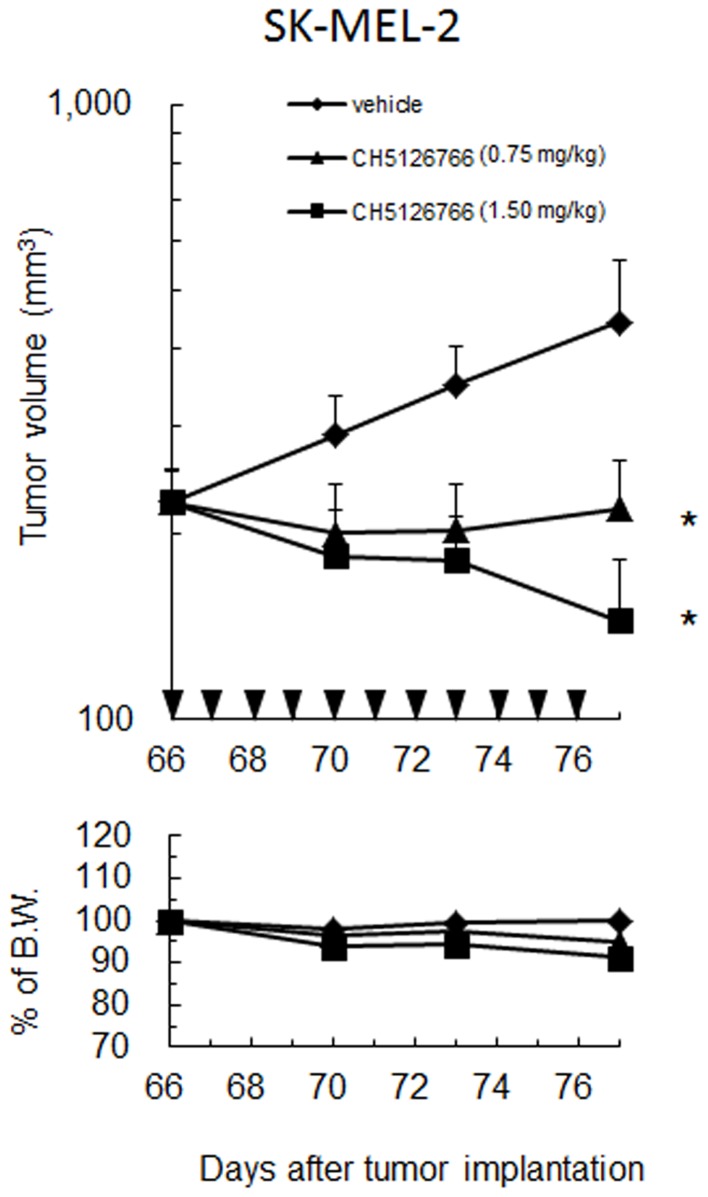
Antitumor activity of CH5126766 in a xenograft model of SK-MEL-2 cells. No animals died or had more than 10% of their body weight gain or loss from baseline in these experiments. Vehicle or CH5126766 was administered orally every day for eleven days. Statistical analysis was conducted by using Dunnett test. *, P<0.05. ▾; Administration of vehicle or CH5126766

Previous reports have shown that ERK feedback inhibition targeted both CRAF/Raf-1 and BRAF [21]–[23]. To determine the key molecule to accumulate phospho-MEK by the MEK inhibitor treatment, siRNAs were used to knock down BRAF or CRAF expression in SK-MEL-2 cells. Phospho-MEK and phospho-ERK levels were reduced in cells treated with siCRAF (Fig. 4A). On the other hand, phospho-MEK and phospho-ERK levels were unaffected in cells treated with siBRAF (Fig. 4A). These results indicate that CRAF, but not BRAF phosphorylates MEK in NRAS-mutated cells. The knockdown of CRAF reduced phospho-MEK accumulation by the MEK inhibitor PD0325901, and the combined knockdown of BRAF and CRAF was also suppressed (Fig. 4B). Additionally, we examined the antitumor effects of CH5126766 in a mouse xenograft model with SK-MEL-2 cells. As shown in Figure 5, CH5126766 suppressed the tumor growth without body weight loss more than 10% in mice.

## Discussion

CH5126766 is a potent and selective dual RAF/MEK inhibitor, which was discovered by the screening to detect p27-inducing agents [18], and is under early clinical trials [24]. In a phase I study with melanoma patients, there were three partial response cases. Interestingly, a genetic abnormality in one melanoma patient was the NRAS mutation, whereas the other two patients harbored the BRAF V600E mutation [24]. A previous study has suggested that cell lines harboring the KRAS mutation such as HCT116 as well as the BRAF mutation are likely to be sensitive to CH5126766 [18], whereas RAS-mutant cancer cells may not be sensitive to other MEK inhibitors. In this study, we also confirmed the antitumor effects of CH5126766 in NRAS-mutated melanoma cells *in vivo* as well as *in vitro*, and also in KRAS-mutated cells *in vitro*.

In the present study, CH5126766 caused regression of tumor without apparent apoptosis *in vitro*, which was consistent with the clinical data that RAF or MEK inhibitors showed quite good response rate reflecting regression of advanced melanoma (4, 6, 7). We hypothesize that RAF or MEK inhibitors might cause cell death *in vivo* due to following mechanisms, such as inhibition of angiogenesis [25] and enhancement of tumor immunity by up-regulation of the CD8^+^T/T_reg_ ratio [26] or down-regulation of PD-L1 [27].

CH5126766 surely suppressed the expression of cyclinD1 and the colony formation more effectively than PD0325901 (Fig. 1D and 1E), even though CH5126766 and PD0325901 equally suppressed phospho-ERK levels in SK-MEL-2 cells (Fig. 1D). Recent reports showed that there was a poor correlation between phospho-ERK inhibition and the anti-proliferative effects of MEK inhibitors in melanoma cells, showing that Ki67, DUSP, Sprouty and phospho-MEK were possible markers for effects of MEK inhibitors [20], [28]. In fact, Ishii *et al*. reported that CH5126766 suppressed phospho-ERK levels to the same degree with PD0325901, but suppressed more effectively the growth of tumors than PD0325901 in xenograft tumor with KRAS mutation [18]. In PD0325901-treated tumors, phospho-MEK was highly induced but not associated with an ERK phosphorylation [18]. In our study, phospho-MEK levels were also significantly increased by PD0325901, and CH5126766 effectively suppressed both MEK and ERK phosphorylation in SK-MEL-2 cells (Fig.1D), which might be one of explanations as to the difference of the efficacy of CH5126766 and PD0325901 (Fig. 1E).

RAS proteins are membrane-bound small G proteins, whereas RAF, MEK, and ERK are cytosolic protein kinases that form a tiered protein kinase cascade downstream of RAS [29]. Recent reports have suggested that CRAF is essential for RAS-driven malignancies such as colon, lung, and epidermal cancers [30]–[33]. In melanoma cells harboring the NRAS mutation, the mutated NRAS protein needs CRAF to activate the MEK/ERK pathway [34], and the BRAF protein is phosphorylated by ERK on S151 near the RAS binding domain, which results in the inhibition of the NRAS/BRAF interaction [35]. Although the knockdown of CRAF decreased phospho-MEK and phospho-ERK levels in this study, the knockdown of BRAF did not affect phospho-MEK and phospho-ERK levels in SK-MEL-2 cells (Fig. 4A). Therefore, CRAF, but not BRAF plays a critical role in mediating RAS signaling and is a suitable therapeutic target in RAS-mutated cells.

As described above, ERK directly phosphorylated BRAF and CRAF resulting in the suppression of their kinase activities [21]–[23]. The MEK inhibitor reactivated RAF proteins in RAS-mutated cells due to the abrogation of the negative feedback pathway, which lead to the accumulation phospho-MEK [36]. The inhibition of RAF reactivation is necessary to abrogate the reactivation of MEK in cells treated with MEK inhibitors. However, combined treatment with PLX4720 and PD0325901 could not completely inhibit MEK activity in NRAS-mutated cells and KRAS-mutated cells (Fig. 2A, 3C), because RAF inhibitor PLX4720 was relatively selective toward BRAF. CH5126766 concurrently suppressed RAF and MEK without the reactivation of RAF and MEK.

Although the BRAF inhibitor or MEK inhibitor improved survival, about 50% of patients treated with the BRAF inhibitor had disease progression within 6 to 7 months after the initiation of the treatment [4], [5]. Several mechanisms of MAPK-dependent and -independent resistance to BRAF inhibitors have been reported [37]. As MAPK-dependent resistance to BRAF inhibitors, up-regulation of COT, secondary mutations in MEK, BRAF splicing variants, and BRAF gene amplification have been reported [37]. As MAPK-independent resistance to BRAF inhibitors, the activation of PI3K-AKT-mTOR pathway has been reported [37]. In melanoma cell lines, which have acquired resistance to the RAF inhibitor, elevated CRAF protein levels accounted for the acquisition of resistance to RAF inhibitor, and CRAF, but not BRAF activated the MEK/ERK pathway [38]. In such cases, CH5126766 may suppress the growth of tumor cells because of inhibition of CRAF activity.

Although the roles of the BRAF V600E mutation in melanoma have been widely studied, several other mutations identified in the BRAF gene are known to cause relatively lower kinase activity [39], [40]. When these mutant BRAF genes were expressed in COS cells, mutant proteins were able to activate the MEK/ERK pathway by directly binding to CRAF [41]. Interestingly, the knockdown of CRAF in melanoma cells with non V600E mutations in BRAF induced apoptosis through a reduction in BAD phosphorylation and Bcl-2 expression [42]. However, the interference of CRAF in melanoma cells harboring the BRAF V600E mutation did not significantly alter their biological properties [43]. Therefore, the inhibition of CRAF is efficacious in melanoma cells harboring the RAS or BRAF mutation, except for V600E. CH5126766 suppressed not only BRAF, but also CRAF kinase activity, and we conclude that CH5126766 is an attractive RAF/MEK inhibitor in RAS-mutated malignant tumor cells including melanoma. We then expect that CH5126766 has a high therapeutic potential with longer survival against RAS-mutated malignant tumors.

## Supporting Information

Figure S1 **Apoptosis analysis in SK-MEL-28 and SK-MEL-2 cells.** SK-MEL-28 and SK-MEL-2 cells were treated with the indicated concentrations of CH5126766 or PD0325901 for 24 h. The percentage of cells in sub-G1 phase was determined by flow cytometry. Data represent means of triplicate with SD indicated.
(TIF)

## References

[1] Flaherty KT (2006) Chemotherapy and targeted therapy combinations in advanced melanoma. Clin Cancer Research 12:2366s–70s. 10.1158/1078-0432.CCR-05-2505 16609060

[2] Hodi FS, O'Day SJ, McDermott DF, Weber RW, Sosman JA, et al (2010) Improved survival with ipilimumab in patients with metastatic melanoma. N Engl J Med 363:711–23. 20525992 10.1056/NEJMoa1003466 PMC3549297

[3] Robert C, Thomas L, Bondarenko I, O'Day S, M D JW, et al (2011) Ipilimumab plus dacarbazine for previously untreated metastatic melanoma. N Engl J Med 364:2517–26. 21639810 10.1056/NEJMoa1104621

[4] Sosman JA, Kim KB, Schuchter L, Gonzalez R, Pavlick AC, et al (2012) Survival in BRAF V600-mutant advanced melanoma treated with vemurafenib. N Engl J Med 366:707–14. 22356324 10.1056/NEJMoa1112302 PMC3724515

[5] Hauschild A, Grob JJ, Demidov LV, Jouary T, Gutzmer R, et al (2012) Dabrafenib in BRAF-mutated metastatic melanoma: a multicentre, open-label, phase 3 randomised controlled trial. Lancet 380:358–65. 22735384 10.1016/S0140-6736(12)60868-X

[6] Flaherty KT, Robert C, Hersey P, Nathan P, Garbe C, et al (2012) Improved survival with MEK inhibition in BRAF-mutated melanoma. N Engl J Med 367:107–14. 22663011 10.1056/NEJMoa1203421

[7] Flaherty KT, Infante JR, Daud A, Gonzalez R, Kefford RF, et al (2012) Combined BRAF and MEK inhibition in melanoma with BRAF V600 mutations. N Engl J Med 367:1694–703. 23020132 10.1056/NEJMoa1210093 PMC3549295

[8] Davies H, Bignell GR, Cox C, Stephens P, Edkins S, et al (2002) Mutations of the BRAF gene in human cancer. Nature 417:949–54. 12068308 10.1038/nature00766

[9] Gray-Schopfer V, Wellbrock C, Marais R (2007) Melanoma biology and new targeted therapy. Nature 445:851–7. 17314971 10.1038/nature05661

[10] Padua RA, Barrass NC, Currie GA (1985) Activation of N-ras in a human melanoma cell line. Mol Cell Biol 5:582–5. 3887133 10.1128/mcb.5.3.582-585.1985 PMC366752

[11] Yamaguchi T, Kakefuda R, Tajima N, Sowa Y, Sakai T (2011) Antitumor activities of JTP-74057 (GSK1120212), a novel MEK1/2 inhibitor, on colorectal cancer cell lines in vitro and in vivo. Int J Oncol 39:23–31. 21523318 10.3892/ijo.2011.1015

[12] Halaban R, Zhang W, Bacchiocchi A, Cheng E, Parisi F, et al (2010) PLX4032, a selective BRAF(V600E) kinase inhibitor, activates the ERK pathway and enhances cell migration and proliferation of BRAF melanoma cells. Pigment Cell Melanoma Res 23:190–200. 20149136 10.1111/j.1755-148X.2010.00685.x PMC2848976

[13] Hatzivassiliou G, Song K, Yen I, Brandhuber BJ, Anderson DJ, et al (2010) RAF inhibitors prime wild-type RAF to activate the MAPK pathway and enhance growth. Nature 464:431–5. 20130576 10.1038/nature08833

[14] Heidorn SJ, Milagre C, Whittaker S, Nourry A, Niculescu-Duvas I, et al (2010) Kinase-dead BRAF and oncogenic RAS cooperate to drive tumor progression through CRAF. Cell 140:209–21. 20141835 10.1016/j.cell.2009.12.040 PMC2872605

[15] Poulikakos PI, Zhang C, Bollag G, Shokat KM, Rosen N (2010) RAF inhibitors transactivate RAF dimers and ERK signalling in cells with wild-type BRAF. Nature 464:427–30. 20179705 10.1038/nature08902 PMC3178447

[16] Tsimberidou AM, Rudek MA, Hong D, Ng CS, Blair J, et al (2010) Phase 1 first-in-human clinical study of S-trans, trans-farnesylthiosalicylic acid (salirasib) in patients with solid tumors. Cancer Chemother Pharmacol 65:235–41. 19484470 10.1007/s00280-009-1027-4

[17] Riely GJ, Johnson ML, Medina C, Rizvi NA, Miller VA, et al (2011) A phase II trial of Salirasib in patients with lung adenocarcinomas with KRAS mutations. J Thorac Oncol 6:1435–7. 21847063 10.1097/JTO.0b013e318223c099

[18] Ishii N, Harada N, Joseph EW, Ohara K, Miura T, et al (2013) Enhanced inhibition of ERK signaling by a novel allosteric MEK inhibitor, CH5126766, that suppresses feedback reactivation of RAF activity. Cancer Res 73:4050–60. 23667175 10.1158/0008-5472.CAN-12-3937 PMC4115369

[19] Lito P, Saborowski A, Yue J, Solomon M, Joseph E, et al (2014) Disruption of CRAF-mediated MEK activation is required for effective MEK Inhibition in KRAS mutant tumors. Cancer Cell 25:697–710. 24746704 10.1016/j.ccr.2014.03.011 PMC4049532

[20] Pratilas CA, Taylor BS, Ye Q, Viale A, Sander C, et al (2009) (V600E)BRAF is associated with disabled feedback inhibition of RAF-MEK signaling and elevated transcriptional output of the pathway. Proc Natl Acad Sci U S A. 106:4519–24. 10.1073/pnas.0900780106 PMC2649208 19251651

[21] Brummer T, Naegele H, Reth M, Misawa Y (2003) Identification of novel ERK-mediated feedback phosphorylation sites at the C-terminus of B-Raf. Oncogene 22:8823–34. 14654779 10.1038/sj.onc.1207185

[22] Dougherty MK, Müller J, Ritt DA, Zhou M, Zhou XZ, et al (2005) Regulation of Raf-1 by direct feedback phosphorylation. Mol Cell 17:215–24. 15664191 10.1016/j.molcel.2004.11.055

[23] Ritt DA, Monson DM, Specht SI, Morrison DK (2010) Impact of feedback phosphorylation and Raf heterodimerization on normal and mutant B-Raf signaling. Mol Cell Biol 30:806–19. 19933846 10.1128/MCB.00569-09 PMC2812223

[24] Martinez-Garcia M, Banerji U, Albanell J, Bahleda R, Dolly S, et al (2012) First-in-human, phase I dose-escalation study of the safety, pharmacokinetics, and pharmacodynamics of RO5126766, a first-in-class dual MEK/RAF inhibitor in patients with solid tumors. Clin Cancer Res 18:4806–19. 22761467 10.1158/1078-0432.CCR-12-0742

[25] Bottos A, Martini M, Di Nicolantonio F, Comunanza V, Maione F, et al (2012) Targeting oncogenic serine/threonine-protein kinase BRAF in cancer cells inhibits angiogenesis and abrogates hypoxia. Proc Natl Acad Sci U S A 109:E353–9. 22203991 10.1073/pnas.1105026109 PMC3277561

[26] Knight DA, Ngiow SF, Li M, Parmenter T, Mok S, et al (2013) Host immunity contributes to the anti-melanoma activity of BRAF inhibitors. J Clin Invest 123:1371–81. 23454771 10.1172/JCI66236 PMC3582139

[27] Jiang X, Zhou J, Giobbie-Hurder A, Wargo J, Hodi FS (2013) The activation of MAPK in melanoma cells resistant to BRAF inhibition promotes PD-L1 expression that is reversible by MEK and PI3K inhibition. Clin Cancer Res 19:598–609. 23095323 10.1158/1078-0432.CCR-12-2731

[28] Smalley KS, Contractor R, Haass NK, Lee JT, Nathanson KL, et al (2007) Ki67 expression levels are a better marker of reduced melanoma growth following MEK inhibitor treatment than phospho-ERK levels. Br J Cancer 96:45–9. 10.1038/sj.bjc.6603596 PMC2360037 17245336

[29] Wellbrock C, Karasarides M, Marais R (2004) The RAF proteins take centre stage. Nat Rev Mol Cell Biol 5:875–85. 15520807 10.1038/nrm1498

[30] Blasco RB, Francoz S, Santamaría D, Cañamero M, Dubus P, et al (2011) c-Raf, but not B-Raf, is essential for development of K-Ras oncogene-driven non-small cell lung carcinoma. Cancer Cell 19:652–63. 21514245 10.1016/j.ccr.2011.04.002 PMC4854330

[31] Karreth FA, Frese KK, DeNicola GM, Baccarini M, Tuveson DA (2011) C-Raf is required for the initiation of lung cancer by K-Ras(G12D). Cancer Discov 1:128–36. 22043453 10.1158/2159-8290.CD-10-0044 PMC3203527

[32] Haigis KM, Kendall KR, Wang Y, Cheung A, Haigis MC, et al (2008) Differential effects of oncogenic K-Ras and N-Ras on proliferation, differentiation and tumor progression in the colon. Nat Genet 40:600–8. 18372904 10.1038/ngXXXX PMC2410301

[33] Ehrenreiter K, Kern F, Velamoor V, Meissl K, Galabova-Kovacs G, et al (2009) Raf-1 addiction in Ras-induced skin carcinogenesis. Cancer Cell 16:149–60. 19647225 10.1016/j.ccr.2009.06.008

[34] Dumaz N, Hayward R, Martin J, Ogilvie L, Hedley D, et al (2006) In melanoma, RAS mutations are accompanied by switching signaling from BRAF to CRAF and disrupted cyclic AMP signaling. Cancer Res 66:9483–91. 17018604 10.1158/0008-5472.CAN-05-4227

[35] Marquette A, André J, Bagot M, Bensussan A, Dumaz N (2011) ERK and PDE4 cooperate to induce RAF isoform switching in melanoma. Nat Struct Mol Biol 18:584–91. 21478863 10.1038/nsmb.2022

[36] Friday BB, Yu C, Dy GK, Smith PD, Wang L, et al (2008) BRAF V600E disrupts AZD6244-induced abrogation of negative feedback pathways between extracellular signal-regulated kinase and Raf proteins. Cancer Res 68:6145–53. 18676837 10.1158/0008-5472.CAN-08-1430

[37] Sullivan RJ, Flaherty KT (2013) Resistance to BRAF-targeted therapy in melanoma. Eur J Cancer. 49:1297–304. 10.1016/j.ejca.2012.11.019 23290787

[38] Montagut C, Sharma SV, Shioda T, McDermott U, Ulman M, et al (2008) Elevated CRAF as a potential mechanism of acquired resistance to BRAF inhibition in melanoma. Cancer Res 68:4853–61. 18559533 10.1158/0008-5472.CAN-07-6787 PMC2692356

[39] Garnett MJ, Marais R (2004) Guilty as charged: B-RAF is a human oncogene. Cancer Cell 6:313–9. 15488754 10.1016/j.ccr.2004.09.022

[40] Wan PT, Garnett MJ, Roe SM, Lee S, Niculescu-Duvaz D, et al (2004) Mechanism of activation of the RAF-ERK signaling pathway by oncogenic mutations of B-RAF. Cell 116:855–67. 15035987 10.1016/s0092-8674(04)00215-6

[41] Garnett MJ, Rana S, Paterson H, Barford D, Marais R (2005) Wild-type and mutant B-RAF activate C-RAF through distinct mechanisms involving heterodimerization. Mol Cell 20:963–9. 16364920 10.1016/j.molcel.2005.10.022

[42] Smalley KS, Xiao M, Villanueva J, Nguyen TK, Flaherty KT, et al (2009) CRAF inhibition induces apoptosis in melanoma cells with non-V600E BRAF mutations. Oncogene 28:85–94. 18794803 10.1038/onc.2008.362 PMC2898184

[43] Hingorani SR, Jacobetz MA, Robertson GP, Herlyn M, Tuveson DA (2003) Suppression of BRAF(V599E) in human melanoma abrogates transformation. Cancer Res 63:5198–202. 14500344

