# Supplementary figures and images for: The Dual RAF/MEK Inhibitor CH5126766/RO5126766 May Be a Potential Therapy for RAS-Mutated Tumor Cells
Source: PLoS One. 2014 Nov 25;9(11):e113217. doi: 10.1371/journal.pone.0113217 (PMC4244135; doi:10.1371/journal.pone.0113217)

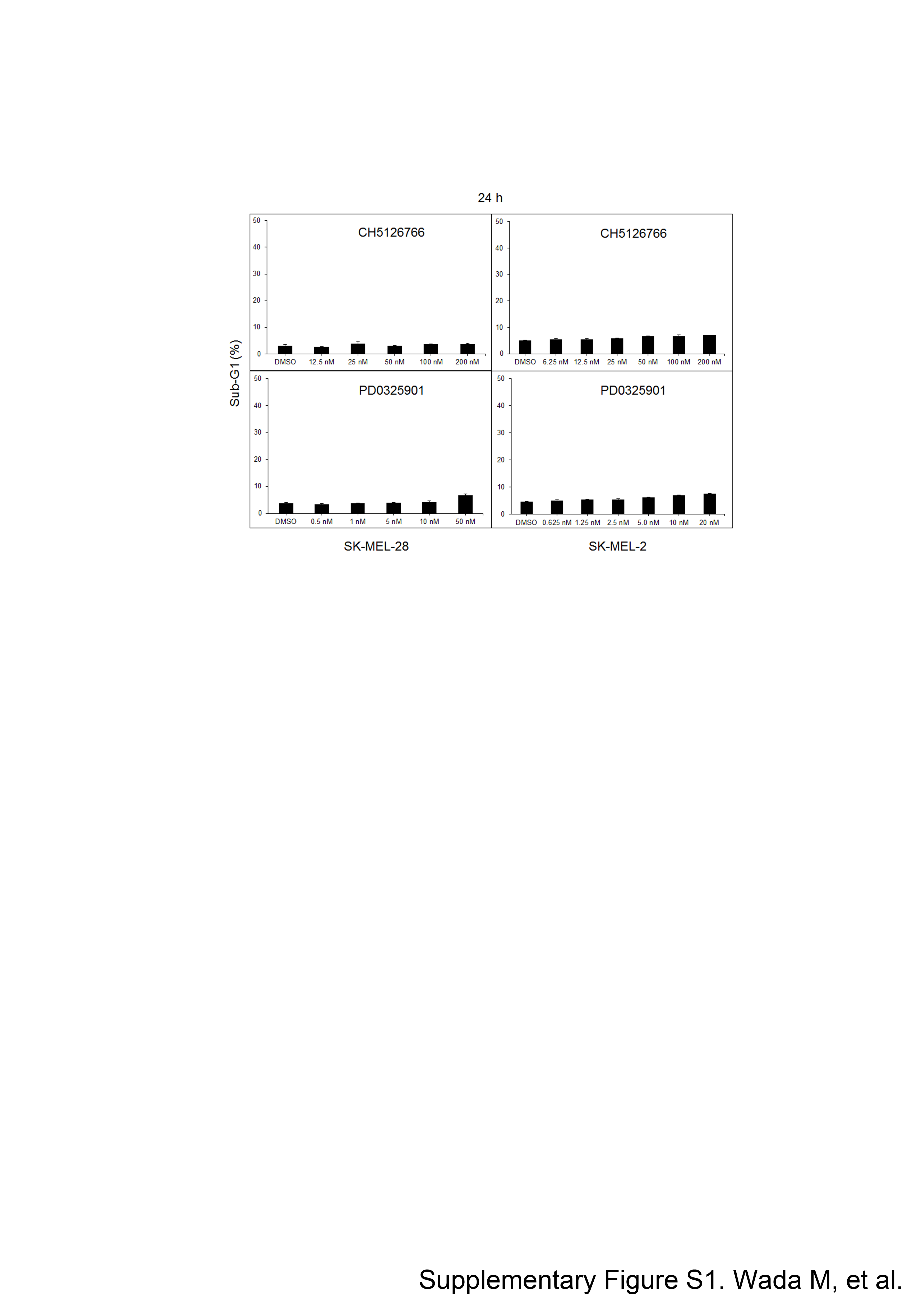

Supplement: Figure S1 — Apoptosis analysis in SK-MEL-28 and SK-MEL-2 cells. SK-MEL-28 and SK-MEL-2 cells were treated with the indicated concentrations of CH5126766 or PD0325901 for 24 h. The percentage of cells in sub-G1 phase was determined by flow cytometry. Data represent means of triplicate with SD indicated. (TIF) [file pone.0113217.s001.tif]
